# Near-maximally swimming schoolmaster snapper (*Lutjanus apodus*) have a greater metabolic capacity, and only a slightly lower thermal tolerance, than when tested at rest

**DOI:** 10.1242/jeb.249273

**Published:** 2024-11-19

**Authors:** Julie J. H. Nati, Peter Malorey, Anthony K. Gamperl

**Affiliations:** ^1^ Department of Ocean Sciences, Memorial University of Newfoundland and Labrador, St. Johns, NL A1C 5S7, Canada; ^2^ School of Biological and Marine Sciences, University of Plymouth, Plymouth PL4 8AA, UK

**Keywords:** Critical thermal maximum, Swim performance, Temperature, Metabolism, Climate change, Tropical fish

## Abstract

To assess the relationship among various measures of thermal tolerance and performance suggested for use in fish, we determined the critical thermal maximum (CT_max_), critical swimming speed (*U*_crit_), maximum thermal tolerance while swimming [CTS_max_] and realistic aerobic scope (AS_R_) of juvenile schoolmaster snapper (*Lutjanus apodus*). Their CTS_max_ (37.5±0.1°C) was only slightly lower than their CT_max_ (38.9±0.1°C) and this is probably because their maximum metabolic rate (MMR) and AS_R_ during the former test were ∼42 and 65% higher, respectively. Furthermore, we did not observe a transition to unsteady (i.e. anaerobically fueled) swimming in the CTS_max_ test as we did in the *U*_crit_ protocol. These data strongly suggest that thermal tolerance tests on fishes whose lifestyle involves schooling or sustained activity should be performed at ecologically relevant swimming speeds. Our results do not support the hypothesis that failure during a CTS_max_ test is due to a fish's inability to meet its tissue oxygen demands.

## Teaser

**Summary:** The upper thermal tolerance, and metabolic capacity, of schoolmaster snapper are different when resting (inactive) versus when swimming close to their maximum velocity.

## INTRODUCTION

Projections are that average ocean temperatures could increase by up to 4°C by 2100, and that this warming will be associated with an increased frequency of extreme and acute warming events (i.e. heat waves) at local and regional scales (Collins et al., 2019; Cooley et al., 2022; Frölicher et al., 2018; Frölicher, 2019; Frölicher and Laufkötter, 2018; Oliver et al., 2018). Given that not all fish species will be able to shift their distribution by moving to higher latitudes or deeper depths (Campana et al., 2020; Perry et al., 2005; Rijnsdorp et al., 2009; Simpson et al., 2011), it is imperative that we understand how increases in temperature will impact the physiology of ecologically and economically important fish species if management and conservation efforts are to be effective (e.g. Little et al., 2020; Metcalfe et al., 2012; Seebacher et al., 2023).

**List of abbreviations**

AS: aerobic scope
AS_R_: realistic aerobic scope
BL: body length
CT_max_: critical thermal maximum
CTS_max_: critical thermal maximum when swimming
*f*_H_: heart rate
MMR: maximum metabolic rate
*Ṁ*_O_2__: oxygen uptake
RMR: resting metabolic rate
SMR: standard metabolic rate
*U*_crit_: critical swimming speed
*U*_GT_: gait transition swimming speed

The most commonly used method to assess the thermal tolerance of fishes is the critical thermal maximum (CT_max_) protocol, which involves acutely warming the water until loss of equilibrium (LOE) (Lutterschmidt and Hutchison, 1997). However, the CT_max_ test is performed on resting (i.e. inactive) fish with LOE as the endpoint, and thus, this measure of acute thermal tolerance may not accurately predict that of fish which typically swim for prolonged periods of time. Recently, a critical thermal maximum when swimming (CTS_max_) test, where fish swim at speeds close to their maximum metabolic rate (MMR, i.e. aerobic capacity) while warmed until fatigue has been used to determine the thermal tolerance of two freshwater tropical fish species [pacu (*Piaractus mesopotamicus*) and tilapia (*Oreochromis niloticus*); Blasco et al., 2020, 2022] and a temperate marine species [European sea bass (*Dicentrarchus labrax*); Nati et al., 2023]. Interestingly, these studies report that MMR and aerobic scope (AS) during the CTS_max_ protocol are greater than measured in either a *U*_crit_ test or the CT_max_ test, whereas CTS_max_ is ∼1.5°C (Blasco et al., 2020, 2022) to 4°C (Nati et al., 2023) lower than a species' CT_max_. Furthermore, it is suggested that the transition to ‘burst-and-coast’ swimming just prior to the end of a CTS_max_ test indicates that this metric of thermal tolerance is linked to an inability of the fish to meet tissue oxygen demands. These data have important implications with regard to what factors/mechanisms determine the thermal tolerance of fishes, and what thermal tolerance protocol should be performed on fishes of a particular lifestyle (i.e. sedentary versus active).

Many tropical marine fish species are already living near their upper temperature limits (Munday et al., 2012; Nati et al., 2021; Rummer et al., 2014; Sunday et al., 2011) and data on the thermal biology of such species is limited compared with those from cooler (i.e. temperate or polar) regions. Thus, in this study, we determined the *U*_crit_, CT_max_ and CTS_max_, and metabolic capacity (MMR and AS_R_) during these protocols, using wild juvenile schoolmaster snapper (*Lutjanus apodus* Walbaum 1792) collected in Eleuthera, The Bahamas. Our hypotheses were that: the CTS_max_ of this species would only be slightly less than their CT_max_; that the metabolic capacity (MMR and AS_R_) of fish in the CTS_max_ test would be greater than in the other two test protocols; and that the fish would show a gate transition (i.e. to ‘burst-and-coast’ swimming) prior to reaching their CTS_max_.

## MATERIALS AND METHODS

### Ethical approval

All research was conducted under a permit issued by the Departments of Environmental Planning and Protection and Marine Resources (BS-2022-873637) of The Bahamas. Furthermore, this research was approved by Memorial University of Newfoundland and Labrador (protocol 22-01-KG), and performed in accordance with the Canadian Council on Animal Care Guidelines on the ‘Care and Use of Fish in Research, Teaching and Testing’ (Canadian Council on Animal Care).

### Animals

The schoolmaster snapper (*Lutjanus apodus*) is an abundant opportunistic predator that is associated with coral reefs in the Caribbean, Gulf of Mexico and northeastern parts of South America, and that is exposed to substantial variations in temperature seasonally and diurnally (in particular as juveniles where they inhabit mangrove forests and creeks; Schneider et al., 2023; Sandrelli et al., 2024). It has also been suggested as a model species for studies of tropical/subtropical reef fish thermal biology and physiology as it inhabits coral reefs as subadults/adults, and is closely related to other high-value snapper species [e.g. mutton snapper, *Lutjanus analis* (Claro et al., 2009); cubera snapper, *Lutjanus cyanopterus* (Motta et al., 2022)].

Juvenile schoolmaster snapper (∼10–12 cm and 30–40 g; see Table 1) were caught on 25–28 February 2023 using baited minnow traps at the Island School (Cape Eleuthera, The Bahamas). These traps were checked regularly and captured specimens were placed in a 1.2 m^3^ (outdoor but under shelter) tank at the Cape Eleuthera Institute (CEI; part of the Island School) that was supplied with flow-through seawater which ranged from ∼25.5 to 27°C daily and had an oxygen content ≥95% air saturation. These fish were held for a minimum of 2–3 days, and were fed daily *ad libitum* with frozen sardines (*Sardinella aurita*). However, fish were not fed within 20 h of the start of an experiment. Below, we describe three different protocols (tests) that were performed on the fish. Note that all fish were naïve when tested; i.e. they were not used in more than one protocol.

**Table 1.**
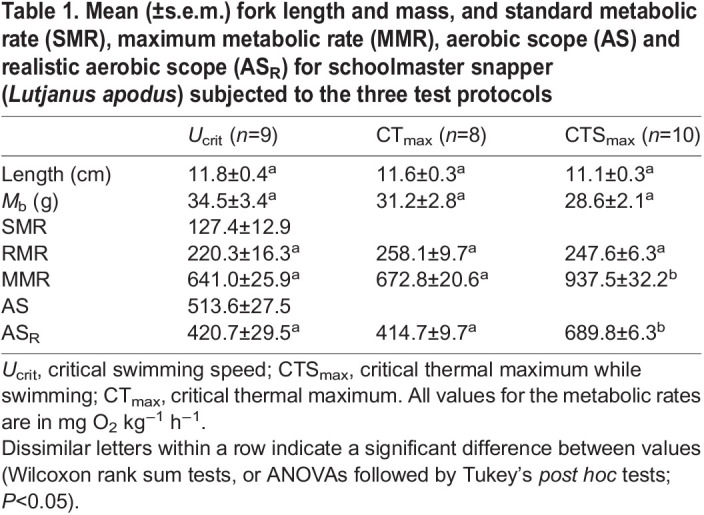
Mean (±s.e.m.) fork length and mass, and standard metabolic rate (SMR), maximum metabolic rate (MMR), aerobic scope (AS) and realistic aerobic scope (AS_R_) for schoolmaster snapper (*Lutjanus apodus*) subjected to the three test protocols

### Swimming performance at acclimation temperature

Fish were measured for mass (to the nearest 0.1 g), fork length, depth and width (to the nearest mm) after light anaesthesia (0.2 g l^−1^ tricaine methansulfonate, TMS; Syndel Laboratories Ltd., Qualicum Beach, BC, Canada); the latter measurements were recorded so that all swimming speeds could be adjusted for the effect of solid blocking (Bell and Terhune, 1970; Kline et al., 2015).

Each fish (*n*=9) was then placed into one of two 3.9 l Blazka swim-tunnel respirometers with a 6.8 cm internal diameter and 31.7 cm swimming section (Technical Services, Memorial University of Newfoundland and Labrador) supplied with a constant flow (∼ 2 liters min^−1^) of aerated seawater at 26°C, and allowed to recover overnight with a gentle current corresponding to 1.0 BL s^−1^. At this velocity, the water in the swim tunnel was adequately mixed and fish would orient into the current, but did not swim [i.e. this allowed for measurements of resting metabolic rate (RMR)]. With this setup, two fish were tested at the same time.

The next morning (at ∼08:00 h) (∼36 h after the fish were last fed), 2–3 measurements of oxygen consumption were taken at 1.0 BL s^−1^. After these measurements of RMR were completed, the current speed was increased by 0.25 BL s^−1^ every 15 min until the fish fatigued and stopped swimming [i.e. a critical swimming speed (*U*_crit_) protocol was performed] (Brett, 1964). Note that these swimming speeds were based on a custom calibration and took the solid blocking effect into account (see below). The speed at which each fish started to ‘burst-and-coast’ swim (called the speed of gait transition, *U*_GT_) was recorded for each fish, and fatigue was defined as the inability of the fish to free itself from the rear grid of the swim-tunnel for 10 s. After the fish fatigued, the current in the tunnel was returned to 1.0 BL s^−1^, and they were allowed to recover for 3–5 h before being returned to the area where they were caught.

The respirometers/swim tunnels were submerged in a rectangular tank filled with temperature-controlled and aerated seawater to a depth of 25 cm, and water was circulated through the tunnels by a 5 liter min^−1^ Eheim submersible pump (Model Universal 200; Deizisau, Germany). The temperature of the water in the tank was controlled by an Isotemp circulating water bath (Model 4100 R35 HS; Fisher Scientific, Suwanee, GA, USA) connected to a titanium coil that was submerged in the water. The current velocity in these respirometers was generated by a Little Giant^®^ submersible pump (Model 3E-34N) fitted on one end, and pump speed was controlled by Variac^®^ variable voltage transformers (Model 3PN1010B-DAM; ISE Ltd, Cleveland OH, USA). We used a GoPro Hero 9 video camera (1080P and 60 frames per second, and with linear scaling enabled to avoid image distortion) to record small pieces of cooked egg white (<1 mm) in the tunnel at incrementally higher Variac^®^ settings. Then, water velocity at each setting was measured using a time-gating method, DaVinci Resolve software (version 18.6.2, Build 2 in Windows 10; Black Magic Design, Fremont, CA, USA) and a h:min:s:ms overlay. This resulted in a custom calibration between water velocity and Variac® setting for each swim tunnel.

Swimming velocity was corrected for the solid blocking effect of the fish (Bell and Terhune, 1970; Kline et al., 2015) using the formula:
(1)

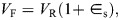
where *V*_F_ is water velocity at the position of the fish's maximum girth, *V*_R_ is water velocity at the rear of the flume and ∈_s_ is the error due to solid blocking. With ∈_s_ calculated as:
(2)

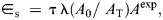
where τ is a dimensionless factor for tunnel cross-section (0.8) and λ is a factor (coefficient) related to the shape of the fish calculated as total body length divided by the total body thickness (Fulton, 2007); *A*_0_ is the cross-sectional area of the fish, and was calculated as 0.25 *G*^2^ π^−1^ (where *G* is girth of the fish, and *G* π^−1^ is thickness); *A*_T_ is the cross-sectional area of the swimming chamber calculated as πr^2^, where *r* is radius and was 3.4 cm, and *A*^exp^ is the fractional area exponent (1.5; Kline et al., 2015).

The fish's critical swim speed (*U*_crit_; in cm s^−1^) was calculated as in Brett (1964):
(3)

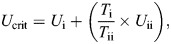
where *U*_i_ is the highest velocity that the fish swam for the entire velocity step; *T*_i_ is the time to fatigue at the last velocity (min); *T*_ii_ is the interval length (15 mins); and *U*_ii_ is the velocity increment (cm s^−1^).

Oxygen uptake (*Ṁ*_O_2__, in mg O_2_ kg^−1^ h^−1^) was measured at each swimming step between 1.0 BL s^−1^ and fatigue using intermittent stop–flow respirometry (Clark et al., 2013; Killen et al., 2021; Rodgers et al., 2016; Steffensen, 1989; Svendsen et al., 2016). Water oxygen concentration was recorded continuously (i.e. every 10 s) using a fiberoptic dipping probe and a 4-channel Witrox^®^ oxygen meter (Loligo^®^ Systems, Viborg, Denmark), and a computer running AutoResp^®^ Software (version 2.3.0; LoligoSystems). These measurements began 5 min after each increase in velocity, included a 2 min ‘wait’ period, and continued until the *r*^2^ of the linear decline in water O_2_ was >0.95. The slope of this relationship was then adjusted for background levels of respiration (i.e. recorded in the tunnel with no fish). These values were quite low (∼1–2%) as the respirometers were cleaned between each *U*_crit_ test and bleached periodically. The fish's *Ṁ*_O_2__ was then calculated as:
(4)

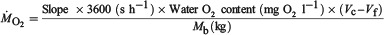
(McKenzie et al., 2003, 2007), where *M*_b_ is fish body mass, *V*_c_ and *V*_f_ are the volume of the swim tunnel and fish, respectively. *V*_f_ was calculated assuming that 1 g of mass=1 ml of water.

For each fish, back extrapolation of the swimming speed–*Ṁ*_O_2__ relationship to 0 BL s^−1^ was used to calculate the fish's standard metabolic rate (SMR), and the highest metabolic rate measured during the *U*_crit_ protocol was recorded as its maximum metabolic rate (MMR). Aerobic scope (AS) was calculated as MMR–SMR and realistic aerobic scope (AS_R_) was calculated as MMR–RMR (Norin et al., 2019; Porter and Gamperl, 2023).

### CT_max_ protocol

Individual fish (*n*=8) were placed in the swim tunnels at 1.0 BL s^−1^ and allowed to recover overnight at ∼26°C. The next morning 2–3 RMR measurements were taken and then the water in the swim tunnel was warmed at 2°C h^−1^ until the fish lost equilibrium; this temperature was recorded as the fish's CT_max_. This rate of heating is consistent with that seen during diurnal changes in temperature in mangrove creeks and a shallow patch reefs in the vicinity of the Island School (Sandrelli et al., 2024) and thus ecologically relevant. Furthermore, this is the same rate of heating used in the CTS_max_ and CT_max_ protocols in Blasco et al. (2020, 2022) and Nati et al. (2023). Measurements of *Ṁ*_O_2__ were taken at each 1°C increase in temperature, and MMR and AS_R_ were calculated as above. Thereafter, the water temperature was quickly returned to 26°C, the fish were allowed to recover for several hours and they were returned to the ocean.

### Critical thermal maximum while swimming (CTS_max_) protocol

The protocol used was similar to that described by Blasco et al. (2017, 2020) and Nati et al. (2023). Control trials (using *n*=6 fish) were first run to establish that the fish could swim for at least 6 h at 3.0 BL s^−1^ [79.9% of the mean U_crit_ and 82.1% of the lowest recorded *U*_crit_] at their acclimation temperature of 26°C (see above and the Results). Then, experiments were performed with an additional 10 fish, where the water velocity was slowly (i.e. over ∼10 min) increased to 3 BL s^−1^, and water temperature was increased by 2°C h^−1^ until the fish fatigued at this constant velocity. After the fish fatigued, the current in the tunnel was reduced to 1.0 BL s^−1^ and water temperature was decreased rapidly by filling the rectangular tank with water from the seawater supply system (∼26°C). After several hours at 26°C, these fish were released in the area where they were captured.

Measurements of *Ṁ*_O_2__ (i.e. RMR) were recorded at 1.0 BL s^−1^ (i.e. before the fish began to swim) in both trials, and at 30 min intervals when the fish were swimming constantly for 6 h at 26°C. They were taken at 1°C intervals (approx. every 30 min) as the fish were being warmed until fatigue. MMR and AS_R_ were calculated as above, with MMR recorded as the highest *Ṁ*_O_2__ value for each fish. The temperature at which the fish stopped swimming was recorded as their CTS_max_.

### Statistical analyses

All statistical analyses were performed using R v. 4.0.2 (r-project.org), with the level of significance set at *P*<0.05. Grubbs and Shapiro tests were used to detect outliers (although none were identified) and to ensure the data were normally distributed, respectively. Statistical differences between CTS_max_ and CT_max_ were identified using a Wilcoxon rank sum test as CTS_max_ was not normally distributed, whereas metabolic parameters measured using the three protocols were examined using one-way ANOVAs followed by Tukey's *post hoc* tests. All data in the text, and in figures and tables, are means±s.e.m.

## RESULTS AND DISCUSSION

**Fig. 1.**
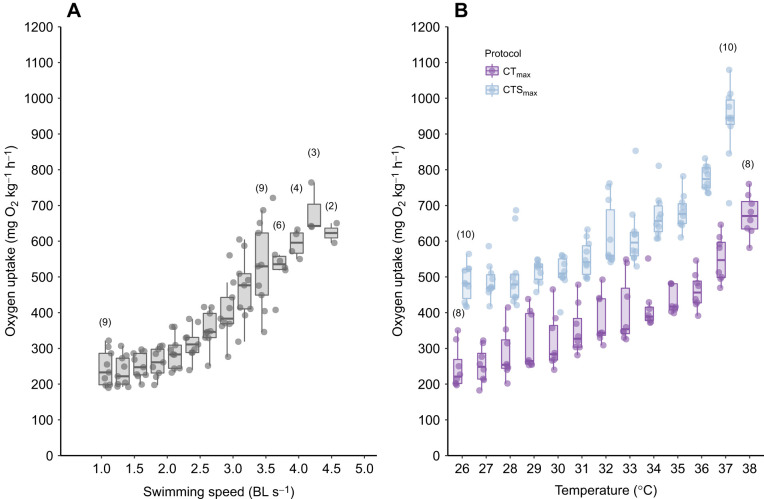
**Oxygen consumption of schoolmaster snapper (*****Lutjanus apodus*****) when subjected to various performance tests**. (A) A critical swim speed (*U*_crit_) test at 26°C (*n*=9). (B) Critical thermal maximum (CT_max_; in purple) test or a critical thermal maximum while swimming (CTS_max_, in blue) test with swimming speed set to 3.0 BL s^−1^ (*n*=8 and 10, respectively). The lower and upper box boundaries indicate the 25th and 75th quartiles, respectively, the line inside the box is the median value, and the vertical lines delimit the 10th and 90th percentiles, respectively. In A, the values above the data points indicate that the number of fish still swimming was less than *n*=9. Note that fish (*n*=6) that were swum at 3.0 BL s^−1^ for 6 h at 26°C did not fatigue and had a constant metabolic rate of 450–500 mg O_2_ kg^−1^ h^−1^ (as determined by a one-way ANOVA).

Prior to reaching their *U*_crit_ (3.9±0.1 BL s^−1^) all fish engaged in ‘burst-and-coast’ swimming and the mean *U*_GT_ value was 3.4±0.10 BL s^−1^ (range: 3.2–4 BL s^−1^). RMR was 220.3±16.3 mg O_2_ kg^−1^ h^−1^ at 26°C, ∼1.7-fold higher than the SMR (127.4±12.9 mg O_2_ kg^−1^ h^−1^), and MMR, AS and AS_R_ were 641.0±25.9, 513.6±27.5 and 420.7±29.5 mg O_2_ kg^−1^ h^−1^, respectively (Fig. 1A; Table 1).

The CT_max_ value for schoolmaster snapper was 38.9±0.1°C, and values for MMR and AS_R_ (672.8±20.6 and 414.7±9.7 mg O_2_ kg^−1^ h^−1^) were not significantly different than measured in the *U*_crit_ trial (Table 1, Fig. 1B).

In the control group for the CTS_max_ experiment, all fish swam at 26°C using a steady swimming mode, and the fish's *Ṁ*_O_2__ did not change (∼450–500 mg O_2_ kg^−1^ h^−1^) over the 6 h period. During the CTS_max_ test, *Ṁ*_O_2__ increased in a curvilinear fashion similar to that observed in the *U*_crit_ experiment (Fig. 1). However, MMR was much higher in the CTS_max_ experiment than in both the *U*_crit_ and CT_max_ trials (∼940 versus 640–670 mg O_2_ kg^−1^ h^−1^; Table 1). In fact, *Ṁ*_O_2__ nearly doubled between the start of the trial and when the fish reached its CTS_max_. This resulted in AS_R_ being ∼65% higher in the CTS_max_ trial compared with the other two protocols (Table 1). The CTS_max_ for schoolmaster snapper (i.e. the temperature at which they stopped swimming) was 37.5±0.1°C, only ∼1.4°C lower than measured in the CT_max_ experiment. Importantly, none of the fish engaged in ‘burst-and-coast’ swimming (i.e. there was no gait transition) prior to the fish reaching their upper temperature limit.

In this study, we investigated three hypotheses with respect to how metrics of metabolic capacity and thermal tolerance would differ between the three test protocols (CT_max_, *U*_crit_ and CTS_max_). While the difference between CT_max_ and CTS_max_ (1.4°C) was similar to what has been previously reported for tropical fish species, there was no evidence of ‘burst-and-coast’ swimming in the schoolmaster snapper prior to fatique in the CTS_max_ test.

With regard to the CT_max_ of juveniles of this species, we report a value of 38.9°C. This is approximately 1.5°C less than that reported by Schneider et al. (2023) for similar sized fish (41.3°C). This difference is likely related to the difference in the rate of heating (Bates and Morley, 2020; Kingsolver and Umbanhowar, 2018) used in the two studies: 10°C h^−1^ in Schneider et al. (2023), whereas we used an ecologically relevant heating rate of 2°C h^−1^ (Porter and Gamperl, 2023; Sandrelli et al., 2024). Thus, 39°C should be considered the maximum acute temperature that can be tolerated by juveniles of this species. This is approximately the same temperature as measured for sub-adult (mass: 250 g; fork length: 25.3 cm) fish subjected to the same rate of heating (Sandrelli et al., 2024) and maximum values measured in the mangrove creeks of South Eleuthera in the summer with a diurnal range from 28°C up to ∼37–39°C (Schneider et al., 2023). As the hyperoxia that occurs in this ecosystem during the day does not improve the schoolmaster snapper's CT_max_ (Sandrelli et al., 2024), it appears that peak summer temperatures are already close to what this species can tolerate and that climate change could limit the use of mangove creeks by this species.

We report that the CTS_max_ of schoolmaster snapper was only slightly (1.4°C) below its CT_max_. This value is comparable to the ∼1.5°C reported by Blasco et al. (2020) for pacu and tilapia acclimated to 26°C, but much less than the 4°C reported by Nati et al. (2023) for European sea bass acclimated to 18°C. Collectively, these data indicate that if fishes are exposed to an acute warming episode in their natural habitat, the capacity to perform maximum (or near maximum) aerobic metabolic work becomes limited at temperatures below those at which their survival is threatened. Furthermore, although additional work is needed, it would appear that the difference between CT_max_ and CTS_max_ is less in tropical versus temperate fish species.

MMR was comparable between the *U*_crit_ and CT_max_ tests. However, it was 42% greater at the end of the CTS_max_ test. That MMR was comparable in the *U*_crit_ and CT_max_ tests is in contrast to most other studies that have compared the MMR of fishes using a heating rate of 2°C h^−1^ (e.g. Eisenberg et al., 2024; Gollock et al., 2006; Petersen and Gamperl, 2010; Powell and Gamperl, 2016). However, lower values for MMR are not always reported for fish subjected to a CT_max_ test versus a *U*_crit_ test (Norin et al., 2019). Other studies have compared values for MMR obtained in fish using CTS_max_ versus *U*_crit_ and CTS_max_ versus CT_max_ protocols, and while all studies (including the present one) report that MMR is much greater in fish given a CTS_max_ test, the magnitude of the difference is highly variable. For example, Blasco et al. (2020) reported that MMR was 33 and 53% higher in a CTS_max_ test versus a *U*_crit_ test in Nile tilapia and pacu, respectively, in agreement with this study. In contrast, Nati et al. (2023) reported that MMR was 3-fold higher during a CTS_max_ test in sea bass than when swum to their *U*_crit_. At present, we do not know why the above studies report such different values.

In the *U*_crit_ test, all schoolmaster snapper began to use unsteady (‘burst-and-coast’) swimming before the end of the protocol, and on average, this was ∼0.5 BL s^−1^ before they reached their *U*_crit_ (i.e. fatigued). However, during the CTS_max_ trial, we did not oberve a gait transition for any fish. Previous authors have suggested that the appearance of unsteady swimming (a gait transition) during a CTS_max_ test was linked to an inability of the fish to meet tissue oxygen demands during the combined challenges of exercise and warming, and that this finding was consistent with the oxygen and capacity limited thermal tolerance (OCLTT) hypothesis (Blasco et al., 2020; Nati et al., 2023). Whether a fish begins unsteady swimming, or not, is only circumstantial evidence with regard to whether an oxygen limitation is causing the fish to fatigue as temperature increases. However, the fact that schoolmaster snapper did not show a gait transition during the CTS_max_ challenges that such a relationship exists. It is just as likely that temperature effects on muscle force/power development at temperatures much higher than those to which the fish were acclimated, limited their capacity to swim and resulted in fatigue (e.g. see Gamperl and Syme, 2021).

Clearly, more research needs to be conducted on what limits swimming speed in fish during warming. Measurements of blood oxygen content and cardiac function would be particularly insightful given their recognized importance in determining the temperature-dependent swimming performance of fishes (Mignucci et al., 2021; Steinhausen et al., 2008). In addition, the measurement of these parameters would allow a number of hypotheses to be tested to determine why MMR was so much higher in the CTS_max_ test than in either the CT_max_ or *U*_crit_ tests. These specific hypotheses are: (1) that the difference in MMR between fish in the CTS_max_ and *U*_crit_ tests was due to fish in the former test being able to achieve a higher heart rate than those in the *U*_crit_ test that was performed at acclimation temperature; and (2) that two phenomenon explain the higher MMR in fish given a CTS_max_ versus CT_max_ test. First, fish are stationary (resting) during a CT_max_ test, and so they must rely exclusively on buccal–opercular pumping, and the partial pressure of oxygen in the arterial blood, as well as arterial blood oxygen content, fall as temperature is increased (Keen and Gamperl, 2012). In contrast, fish exposed to a considerable current (as in the CTS_max_ test) would be able to engage in ram ventilation, and this could greatly increase the amount of water flowing over the gills and potentially result in higher values for these parameters at a particular temperature. Second, differences in stroke volume could have also contributed to the difference in MMR between fish exposed to CTS_max_ versus CT_max_ tests. In a CT_max_ test, whereas heart rate (*f*_H_) increases substantially with temperature, stroke volume is unchanged (Farrell, 2009). This is probably because central venous pressure does not change in fish when temperature is acutely increased (Sandblom and Axelsson, 2007; although the data are very limited) and stroke volume is constrained by the decrease in the cardiac cycle's diastolic period (time available for filling) as heart rate increases. In contrast, enhanced venous return results in an increase in central venous pressure when fish swim (Sandblom et al., 2005) and this may potentially allow fish challenged with swimming to increase *S*_V_ (and thus blood oxygen transport) as temperature increases.

This study, and addressing the hypotheses that emerged from this work will provide valuable information on the mechanisms determining differences in the MMR and AS (AS_R_) of fishes given ecologically relevant challenges; especially those which relate to climate change. Furthermore, they will add to the ongoing debate as to whether, to what extent and under which circumstances, the OCLTT concept (Blasco et al., 2020; Ern et al., 2014, 2016; Nati et al., 2023; Wang et al., 2014) and the gill-oxygen limited theory (GOLT; Amarsinghe and Pauly, 2021; Bigman et al., 2023; Pauly, 2021; Skeeles and Clark, 2024) have relevance for the thermal tolerance of fishes. However, an important contribution of this, and other recent papers (e.g. Desforges et al., 2023; Ern et al., 2023; McKenzie et al., 2016; Ørsted et al., 2022; Sandrelli and Gamperl, 2023), is that they highlight that measurements of thermal tolerance must be performed carefully (and be ecologically relevant) if they are to be meaningful and useful in implementing conservation and management strategies to protect fish in this era of climate change. This is key as the individuals who make these decisions are not usually experts in fish thermal biology/physiology and are dependent upon us to provide relevant and accurate data. With regard to values of CTS_max_ versus CT_max_ (present study; Blasco et al., 2020, 2022; Nati et al., 2023), it is apparent that the upper temperature that fish can tolerate is different when measured using these two tests. What is needed now are experiments where the upper thermal tolerance of fishes is measured at a number of swimming speeds up to CTS_max_. For example, it is possible that the benefits of ram ventilation at intermediate swimming speeds may result in an upper thermal tolerance that is even higher than CT_max_; although this is unlikely for schoolmaster snapper as hyperoxia does not change CT_max_ in this species (Sandrelli et al., 2024). Such information is critical if we are to accurately determine the thermal tolerance of fish with different activity levels and/or at different points in their life cycle.
